# Curing the Toxicity of Multi-Walled Carbon Nanotubes through Native Small-molecule Drugs

**DOI:** 10.1038/s41598-017-02770-5

**Published:** 2017-06-06

**Authors:** Wei Qi, Longlong Tian, Wenzhen An, Qiang Wu, Jianli Liu, Can Jiang, Jun Yang, Bing Tang, Yafeng Zhang, Kangjun Xie, Xinling Wang, Zhan Li, Wangsuo Wu

**Affiliations:** 1 School of Chemistry and Chemical Engineering, Huazhong University of Science and Technology, Wuhan City, 430074 Hubei Province P.R. China; 2 Lanzhou University, Lanzhou City, 730000 Gansu Province P.R. China; 3 Lanzhou University Second Hospital, Lanzhou City, 730000 Gansu Province P.R. China; 4 Lanzhou Institute of Chemical Physics, Chinese Academy of Sciences, Lanzhou, 730000 Gansu Province P.R. China; 5 Non-power Nuclear Technology Research & Development Center, Hubei University of Science and Technology, Xianning City, 437000 Hubei Province P.R. China; 6 Institue of Applied and Electromagnetic Engineering, Huazhong University of Science and Technology, Wuhan City, 430074 Hubei Province P.R. China; 7 College of Pharmacy, Xinjiang Medical University, Urumqi, 830011 Xinjiang Province P.R. China

## Abstract

With the development and application of nanotechnology, large amounts of nanoparticles will be potentially released to the environment and possibly cause many severe health problems. Although the toxicity of nanoparticles has been investigated, prevention and treatment of damages caused by nanoparticles have been rarely studied. Therefore, isotope tracing and improved CT imaging techniques were used to investigate the biodistribution influence between oMWCNTs(oxidized multi-walled carbon nanotubes) and 1,2-dioleoyl-sn-glycero-3-phosphocholine (DOPC)/or simvastatin (TD) *in vivo*. What’s more, biochemical indices in plasma and tissue histology were measured to further study therapeutic effects on the damages of oMWCNTs in mice. Isotope tracing and improved CT imaging results showed that low dosages of DOPC and TD didn’t affect the distribution of oMWCNTs in mice; conversely, the distribution and metabolism of DOPC and TD were influenced by oMWCNTs. Moreover, DOPC and/or TD improved the biocompatibility of oMWCNTs in erythrocyte suspension *in vitro*. Biochemical index and histopathological results indicated that DOPC and TD didn’t prevent injuries caused by oMWCNTs effectively. But TD showed a good therapeutic effect for damages. This study is the first to investigate prevention and treatment effects of drugs on damages caused by oMWCNTs and provides new insights and breakthroughs for management of nanoparticles on health hazards.

## Introduction

Carbon nanotubes are new types of materials that have been widely studied and applied in electronic engineering, catalytic materials, environmental engineering, biological medicine and other related fields^1–4^. Particularly, the potential application of carbon nanotubes in drug delivery, tumour probing and imaging has attracted considerable research attention^5–7^. Therefore, carbon nanotubes have been used broadly in various areas such as industrial application, consumer products and so on, moreover, it’s reported that the global production of carbon nanotubes sum up to 100–1000 t/a^8^. The widespread usage of carbon nanotubes will increase their concentration in environment so as to cause diverse ecological problems^9, 10^.

Carbon nanotubes are foreign particulate matter in biological organisms; exposure to nanotubes can cause tissue damage or toxicity^11–13^. The biological toxicity of nanomaterials has been evaluated to determine their safety in various environments, the toxicity of nanomaterials is related to their structure, size, functional groups and mechanism of exposure^14–18^. In addition, different functional groups or structure of nanoparticles have strong interaction each other after entering into body, then leading to more complicated damages or effects on biological organisms. Several examples as follows: Li *et al*. intravenously studied bio-distribution in mice co-exposed to oxidised multi-walled carbon nanotubes (oMWCNTs) and oxidised graphene through isotope tracer technique, and found that the bio-distribution of oxidised graphene was considerably affected by oMWCNTs after co-exposure; both of these compounds cause damages to liver and lung^19^. Wang *et al*. reported that single-walled carbon nanotubes (SWCNTs) accumulated mainly in the liver and kidney when mice were exposed intraperitoneally to hydroxylated SWCNTs; SWCNTs also slightly accumulated in the spleen and lung but eventually excreted mainly from urine within 18 days^20^. Singh *et al*. also reported that ammonium-functionalised water-soluble SWCNTs linked with DTPA-^111^In were excreted mainly *via* renal route without uptake in the liver and spleen after being intravenously injected to mice^21^. These findings indicate that the nature of functionalization on nanotubes is the most influential factor on the behaviour and toxicity of carbon nanotubes in mice. In addition, several studies about the toxicity of nanoparticles to sensitive groups (for example, foetus in the pregnant body) found that oMWCNTs could cross placental barrier into the placenta and cause damages to matrix, and the complication reduces the ability of the placenta to provide nutrition for the offspring and affects foetal development, which could cause a series of pregnancy disorders^22^. But more importantly than all of above, there are many researches about the toxicity and interaction of protein, cell and microorganism basing on carbon nanotubes^23–27^, and find that carbon nanotubes could enter into and destroy cell or microorganism, further induced cell apoptosis or function disorder of microorganism, and revealing that the toxicity mechanism of carbon nanotubes may relate to the interaction of protein and carbon nanotubes. However, it can be seen that almost of all reports are about the toxicity and damage of carbon nanotubes in biology body or others, but there are rare or even not researches about the treatment or prevention effects of drugs on the toxicity or damages caused by nanoparticles.

It is a great severe effect on health after exposure to nanoparticles environment. It’s also widely believed that carbon nanotubes can accumulate in tissues for a long time to cause inflammation or function disorders. Therefore, it can be speculated that the toxicity or damage of carbon nanotubes might be decreased or cured through urgent elimination of the accumulated nanoparticles from the biological body as well as treatment of inflammatory disorders or others. Basing on this speculation, the experiment of curing the toxicity of nanoparticles in mice *in vivo* through small-molecular drugs were designed and carried out in here. DOPC in cell membranes are first damaged when carbon nanomaterials (such as graphene) enter into the biological body. It was found that graphene strongly adsorbs DOPC on the surface *in vitro* to form a compound coated with DOPC^28^. It can be imagined that when nanoparticles enter into biology body, the supplementary extra DOPC will compete with the DOPC of cell membrane to be adsorbed by nanoparticles, and so as to decrease the toxicity of nanoparticles; on the other side, many reports prove that the biocompatibility and toxicity of nanoparticles can be improved after coated with DOPC. It can be imagined that nanoparticles adsorb the free extra DOPC in blood vessel after enter into body, and then the biocompatibility of nanoparticles is improved to make them eliminate from body easily. As well know, statins have obvious effects on regulating blood lipid, delaying the extent of atherosclerosis, anti-inflammation and anti-thrombosis, and thus are commonly used to cardiovascular and cerebrovascular diseases or anti-inflammatory in clinical^29–31^. In addition, one of statins pharmacological effects is widening myocardial tissue clearance according to the view of doctors. Thus, statins may promote the excretion and anti-inflammation of nanoparticles that accumulated in the body to a certain degree, thereby reducing or eliminating tissue inflammation^32–35^. In the present work, therefore, prevention and treatment capability of simvastatin (TD) and DOPC on the toxicity of oMWCNTs in mice was studied firstly. The reliability of isotope tracer technique on the biodistribution of nanoparticles *in vivo* was investigated to further study the biodistribution of oMWCNTs *in vivo*. The improved CT imaging was then used to investigate the distribution of oMWCNTs in mice *in vivo*. For single oMWCNTs no development under CT imaging, Ag/oMWCNTs nanocompounds were synthesised as reported in the literature^36^; this technique improved the CT imaging ability of oMWCNTs to further study their biodistribution in mice. This study provides new breakthrough and reference data for prevention and treatment effects of drugs on damages and toxicity caused by nanoparticles to the biological body.

## Results

### Characterization results of oMWCNTs and Ag/oMWCNTs

The TEM characterisation results showed that oMWCNTs had 1–10 μm length and 10–30 nm diameter. In addition, the TEM images of Ag/oMWCNTs showed that almost all Ag ions were connected to the surface of oMWCNTs, and the Ag/oMWCNTs nanocompounds were synthesised (Figure S1). Figure S2 showed that –COOH and –OH were produced on the surface of oMWCNTs after oxidization. The Raman spectrum showed that the structure of the carbon nanotubes did not change after exposure to oxidation (Figure S3) (detailed information is presented in Supplementary Information).

### Tissue biodistribution

**Figure 1:**
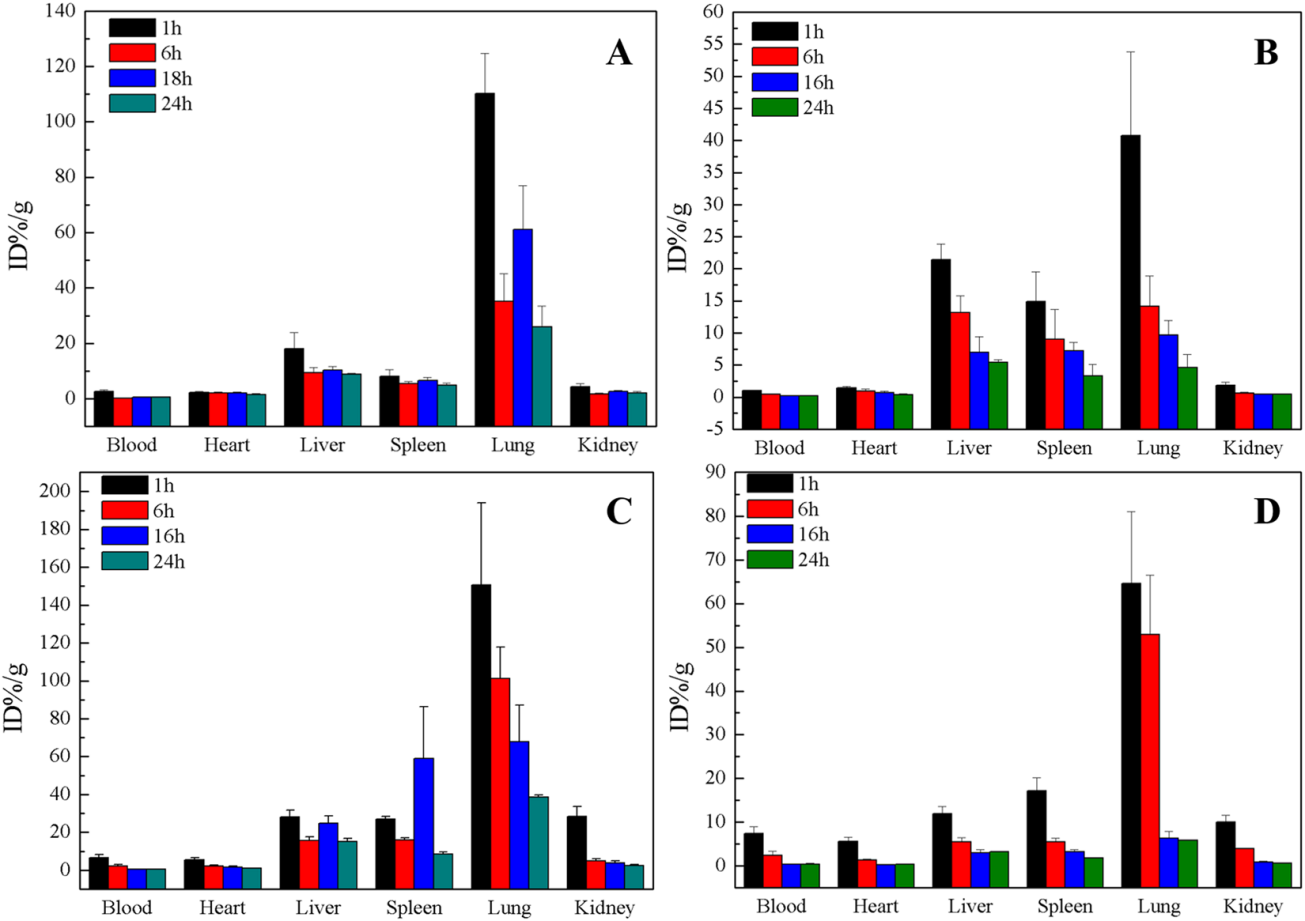
Biodistribution interaction effect of DOPC/or TD on oMWCNTs during 24 h after exposure to mice (**A** and **B** for ^131^I-oMWCNTs + DOPC and ^131^I-DOPC + oMWCNTs respectively, **C** and **D** for ^131^I-oMWCNTs + TD and ^131^I-TD + oMWCNTs, n = 5, +sem).

The effects of DOPC and/or TD on the biodistribution of oMWCNTs in mice were studied through ^131^I isotope tracer technique (Fig. 1). Figure 1A shows the effect of DOPC on the distribution of ^131^I-oMWCNTs *in vivo*, and ^131^I-oMWCNTs mostly accumulated in the lung, liver and spleen of mice; minimal amounts of ^131^I-oMWCNTs also accumulated in the kidney. The radioactive levels of tissues decreased with time, indicating that ^131^I-oMWCNTs could be excreted through metabolism in mice. These results are consistent with those in our previous work^19, 37^. Therefore, the presence of DOPC did not affect the distribution of ^131^I-oMWCNTs in mice. About 1 h after the exposure, ^131^I-DOPC accumulated in the lung, liver and spleen of mice; small amounts were also detected in the heart and kidney. About 24 h after exposure, the accumulated ^131^I-DOPC in tissues decreased with time, but large amounts still remained in the lung, liver and spleen (Fig. 1B). Moreover, the distribution of ^131^I-DOPC coincided with that of oMWCNTs. Figure 1C shows that the presence of TD did not affect the distribution of ^131^I-oMWCNTs in mice. However, high levels of ^131^I-oMWCNTs were found in the blood and kidney 1 h after exposure, indicating that TD could improve the biocompatibility of oMWCNTs and promote their excretion from the body of mice. Figure 1D shows that oMWCNTs greatly affected the distribution of ^131^I-TD in mice, and the distribution states were in accordance with those of oMWCNTs.

**Figure 2:**
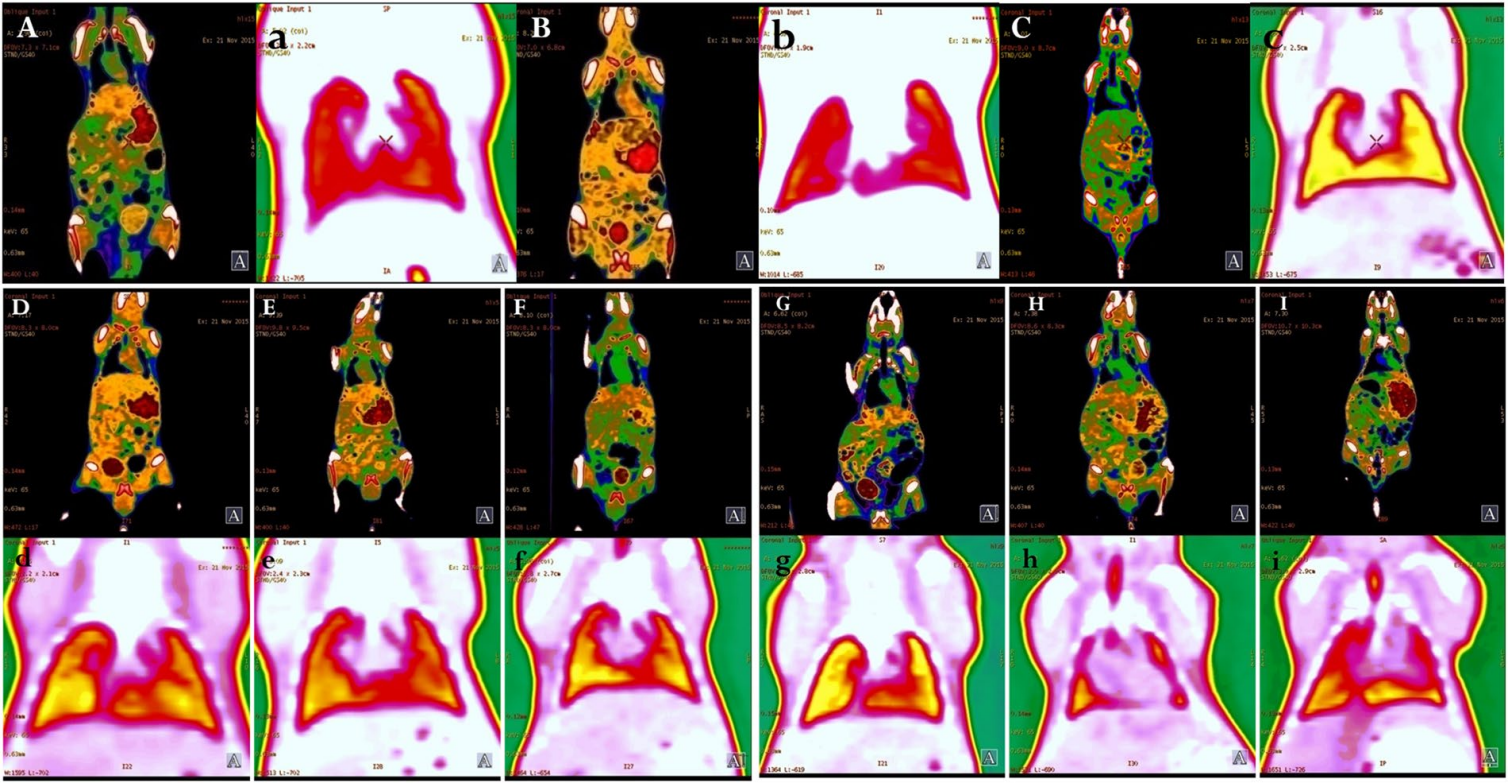
The CT imaging of Ag/oMWCNTs in mice with affection by different dosage of DOPC/or TD (**A**,**B**,**C** is the whole CT imaging of mice after exposure saline solution, single Ag nanoparticles, Ag/oMWCNTs, respectively; (a,b,c) is the lung CT imaging for control, single Ag nanoparticles and Ag/oMWCNTs, respectively; (**D,E,F**) is the whole CT imaging and (d,e,f) is the lung CT imaging of mice after exposure 6.2, 12.4 and 18.6 mg/kg.bw TD to oMWCNTs-model mice, respectively; (**G,H,I**) is the whole CT imaging and (g,h,i) is the lung CT imaging of mice after exposure 6.2, 12.4 and 18.6 mg/kg.bw DOPC to oMWCNTs-model mice, respectively).

The Ag/oMWCNTs nanocompounds were synthesised to improve the CT imaging of oMWCNTs in mice, and the effects of DOPC/or TD on biodistribution of oMWCNTs were further studied *in vivo*. After exposure to single Ag nanoparticles, the spleen and bladder showed obvious uptake with bright imaging, but no evident imaging was observed in the lung (Fig. 2). When the mice were intravenously exposed to Ag/oMWCNTs nanocompounds, only a slight imaging in the spleen and bladder was observed for some free Ag ions. A bright image was observed in the lung tissue through CT imaging, indicating that most of Ag/oMWCNTs were retained in the lung. These results agreed with those of previous radiolabeling studies. Therefore, CT imaging was improved by synthesis of Ag/oMWCNTs nanocompounds to reflect the actual biodistribution of oMWCNTs in mice. The dosage effects of DOPC/or TD on the biodistribution of Ag/oMWCNTs in mice were also investigated through improved CT imaging. After exposing the mice to different dosages of DOPC/or TD (about 6.2, 12.4 and 18.6 mg/kg.bw) with Ag/oMWCNTs nanocompounds (12.4 mg/kg.bw), no obvious difference was observed among the different dosages of TD exposure (Fig. 2). Thus, the dosage of TD did not affect the biodistribution of Ag/oMWCNTs in mice. By contrast, the CT imaging results showed that the imaging of lung tissues decreased with the increase of exposure dosage of DOPC. However, the strong imaging could be seen in the intestinal tract and other tissues with membranes.

### Effect of DOPC/or TD on the toxicity of oMWCNTs in mice

**Figure 3:**
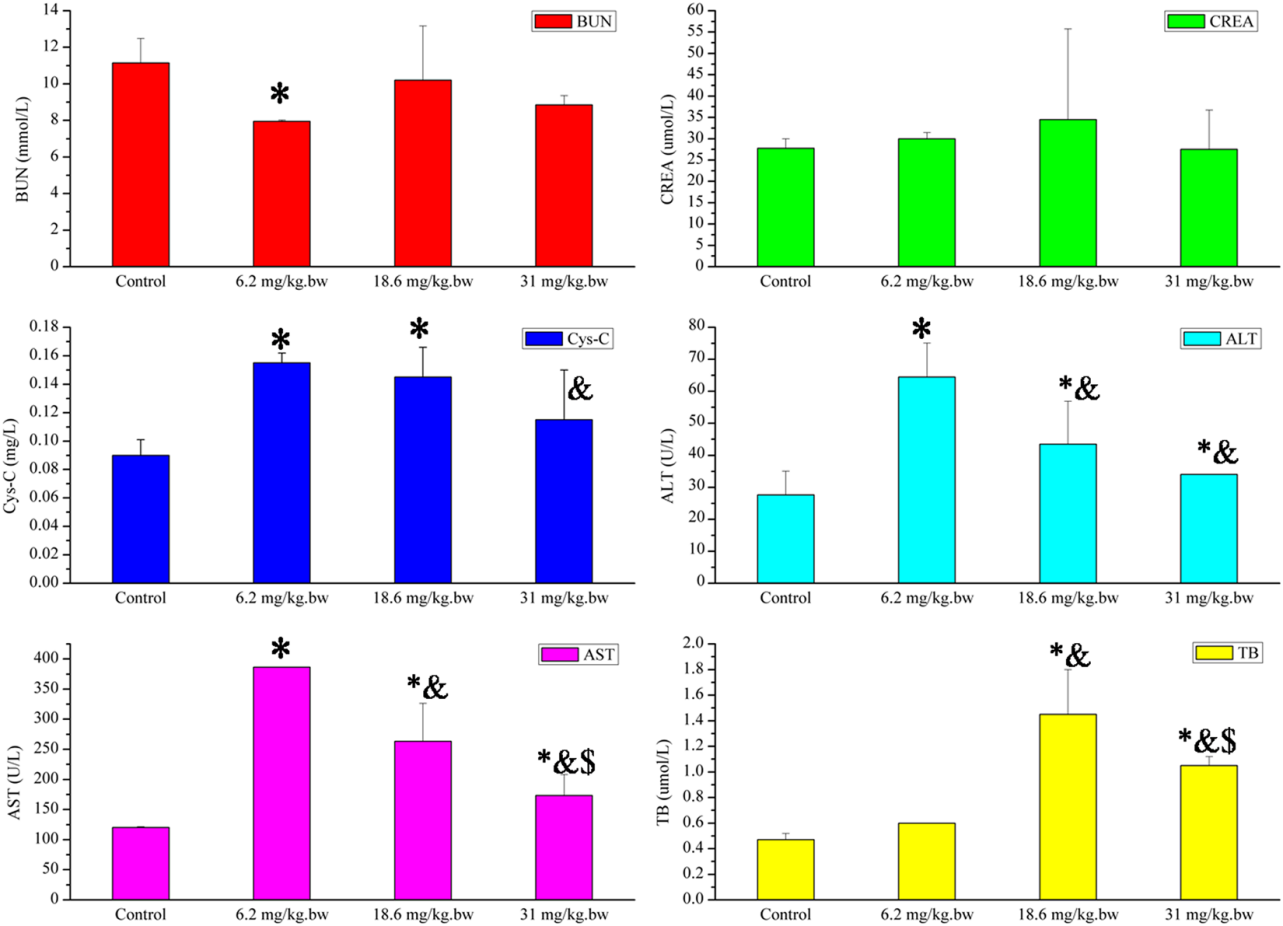
The biochemical indices level of BUN (blood urea nitrogen), CREA (creatinine), AST (aspartate aminotransferase), Cys-C (cystatin C), ALT (alanine aminotransferase) and TB (total bilirubin) change in plasma after exposure different dosages of 6.2, 18.6, 31 mg/kg.bw oMWCNTs to mice (*p < 0.05 for groups *vs*. control group; ^*&*^ *p* < 0.05 for groups vs. 6.2 mg/kg.bw-group; ^*$*^ *p* < 0.05 for groups vs. 12.4 mg/kg.bw-group; n = 5, +sem).

**Figure 4:**
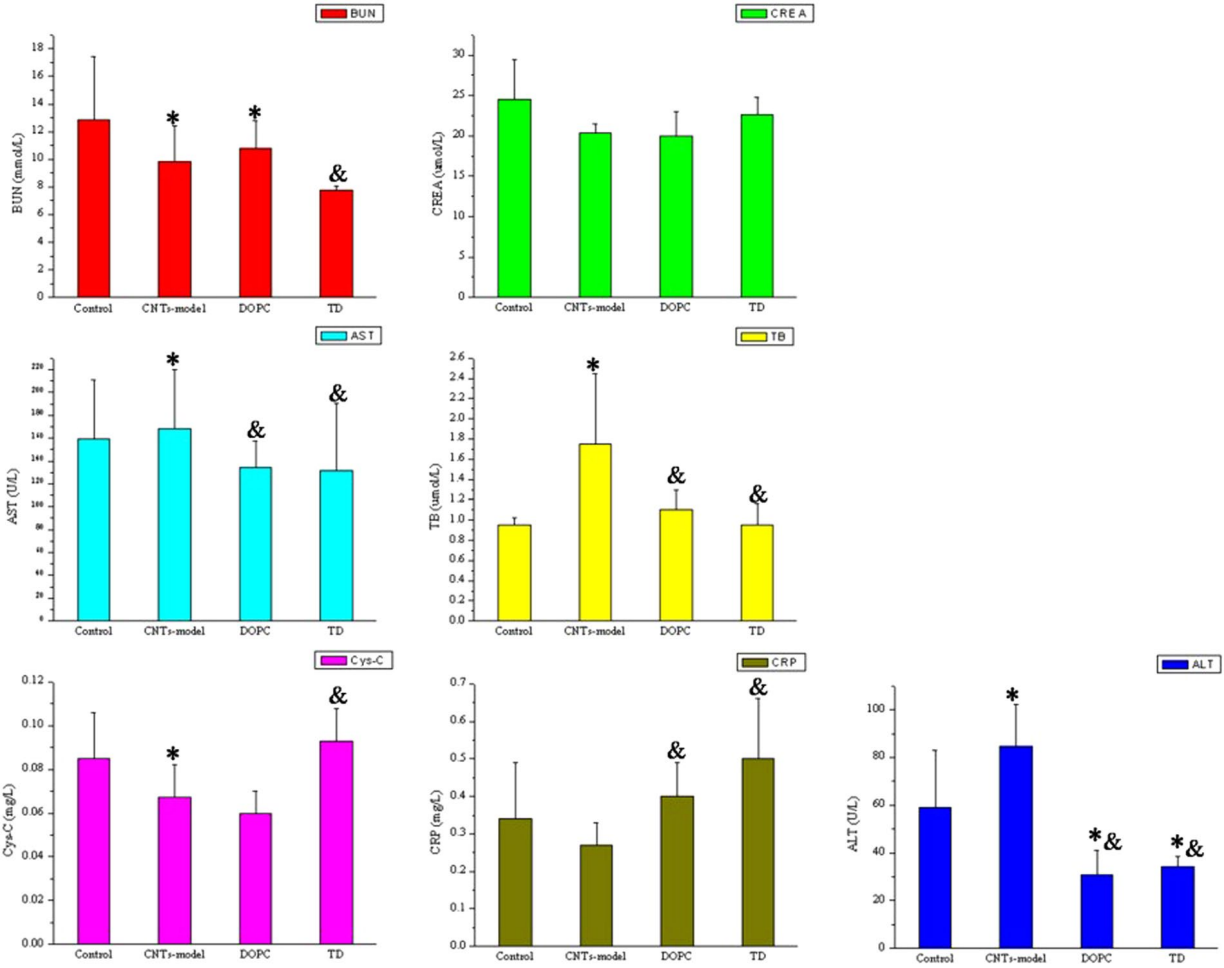
The biochemical indices level of BUN (blood urea nitrogen), CREA (creatinine), AST (aspartate aminotransferase), Cys-C (cystatin C), ALT (alanine aminotransferase), CRP (C-reaction protein) and TB (total bilirubin) change in plasma after exposure DOPC/or TD to oMWCNTs–model mice (*p < 0.05 for groups *vs*. control group; ^*&*^ *p* < 0.05 for groups vs. oMWCNTs-model group; n = 5, +sem).

**Figure 5:**
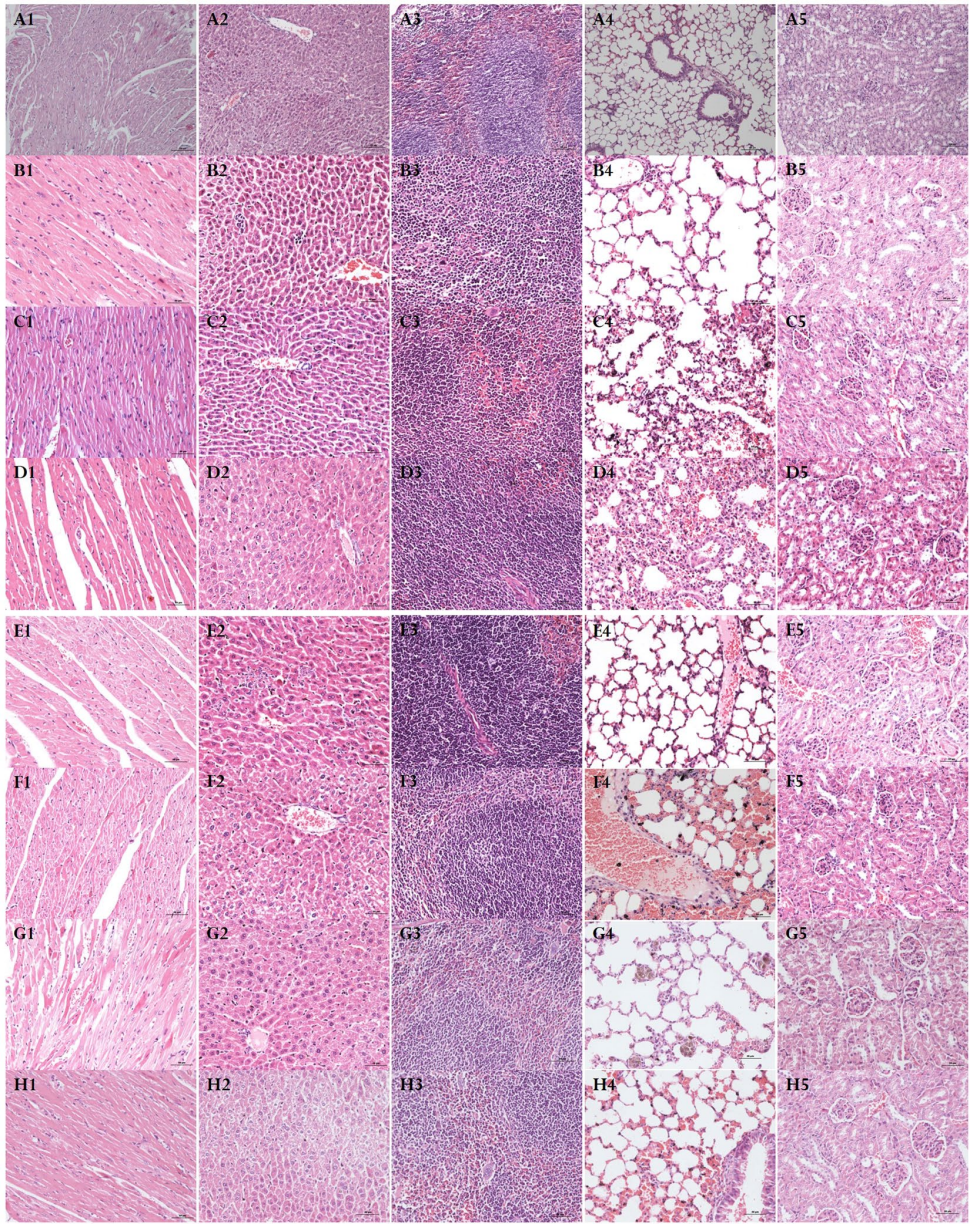
The tissue histology (200×, scale bar is 50 μm for **B–G** groups, and scale bar is 100 μm for **A** group) investigation after exposure different drugs (A1–A5 for tissues of heart, liver, spleen, lung and kidney of control group mice, respectively; B1-B5 for tissues of heart, liver, spleen, lung and kidney of oMWCNTs-model group mice, respectively; C1–C5 for tissues of heart, liver, spleen, lung and kidney of oMWCNTs-model + DOPC group mice, respectively; D1–D5 for tissues of heart, liver, spleen, lung and kidney of oMWCNTs-model + TD group mice, respectively; E1–E5 for tissues of heart, liver, spleen, lung and kidney of TD-model + oMWCNTs group mice, respectively; F1–F5 for tissues of heart, liver, spleen, lung and kidney of DOPC-model + oMWCNTs group mice, respectively; G1–G5 for tissues of heart, liver, spleen, lung and kidney DOPC + oMWCNTs group mice, respectively; H1–H5 for tissues of heart, liver, spleen, lung and kidney of TD + oMWCNTs group mice, respectively).

In this experiment, the model was generated through exposure 12.4 mg.kg.bw oMWCNTs to mice intravenously. Then DOPC/or TD were injected intravenously into mice to observe the treatment effect of DOPC/or TD on the toxicity of oMWCNTs. Figure 3 shows that the damages of oMWCNTs to mice strongly depend on the exposure dosages. The biochemical indices in plasma changed significantly when the exposure dosages increased from 6.2 to 31 mg/kg.bw. However, when the exposure dosage reached 31 mg/kg.bw, the effects of oMWCNTs on mice were reduced because of the agglomeration of oMWCNTs in high concentrations^37^. Therefore, the exposure dosage of oMWCNTs in mice was fixed at 12.4 mg/kg.bw in this experiment. Figure 4 shows that compared with the control groups, the BUN, ALT, AST, Cys-C and TB concentrations in plasma of oMWCNTs-model group mice changed substantially (Fig. 4, *p < 0.05), indicating successful establishment of oMWCNTs-model mice. After exposing the oMWCNTs-model mice to DOPC/or TD, the AST, ALT and TB levels in the plasma of DOPC-exposed mice were lower than those of the oMWCNTs-model group mice and approximate to those of the control group of mice. However, for the TD-exposed group of mice, the BUN, ALT, AST, Cys-C, TB and CRP contents in the plasma were considerably different from those of the oMWCNTs-model group of mice. Most of the measured indices reverted to the normal levels after the oMWCNTs-model group mice was exposed to TD. In addition, the histology of tissues showed the same results (Fig. 5 and Figure S8). Compared with the control group of mice, the histology of oMWCNTs-model group mice showed that the pulmonary alveoli ruptured seriously, the lung tissues bled and the nanoparticles accumulated. The hepatic cords were almost normal, but nanoparticles were present in the liver pathological section. The fibrosis of spleen histology showed that the spleen tissue was damaged. The histology of kidney indicated that parietal cells were damaged or disappeared, and the structure of podocytes and kidney tubules were disordered. However, no evident histological change in the myocardial cell was observed. For the histology of oMWCNTs-model mice exposed to DOPC, the pulmonary alveoli expanded and showed fractures. The arrangement of hepatic cords was slightly disordered with no obvious lesions. No lesions were observed in the histology of the spleen, kidney and heart. For the histology of oMWCNTs-model mice exposed to TD, all tissues were normal except for the slight tissue bleeding of lung tissues and the disordered arrangement of hepatic cords. Therefore, both DOPC/or TD showed a certain treatment effects on the damages caused by oMWCNTs in mice, and TD exhibited improved therapeutic results.

**Figure 6:**
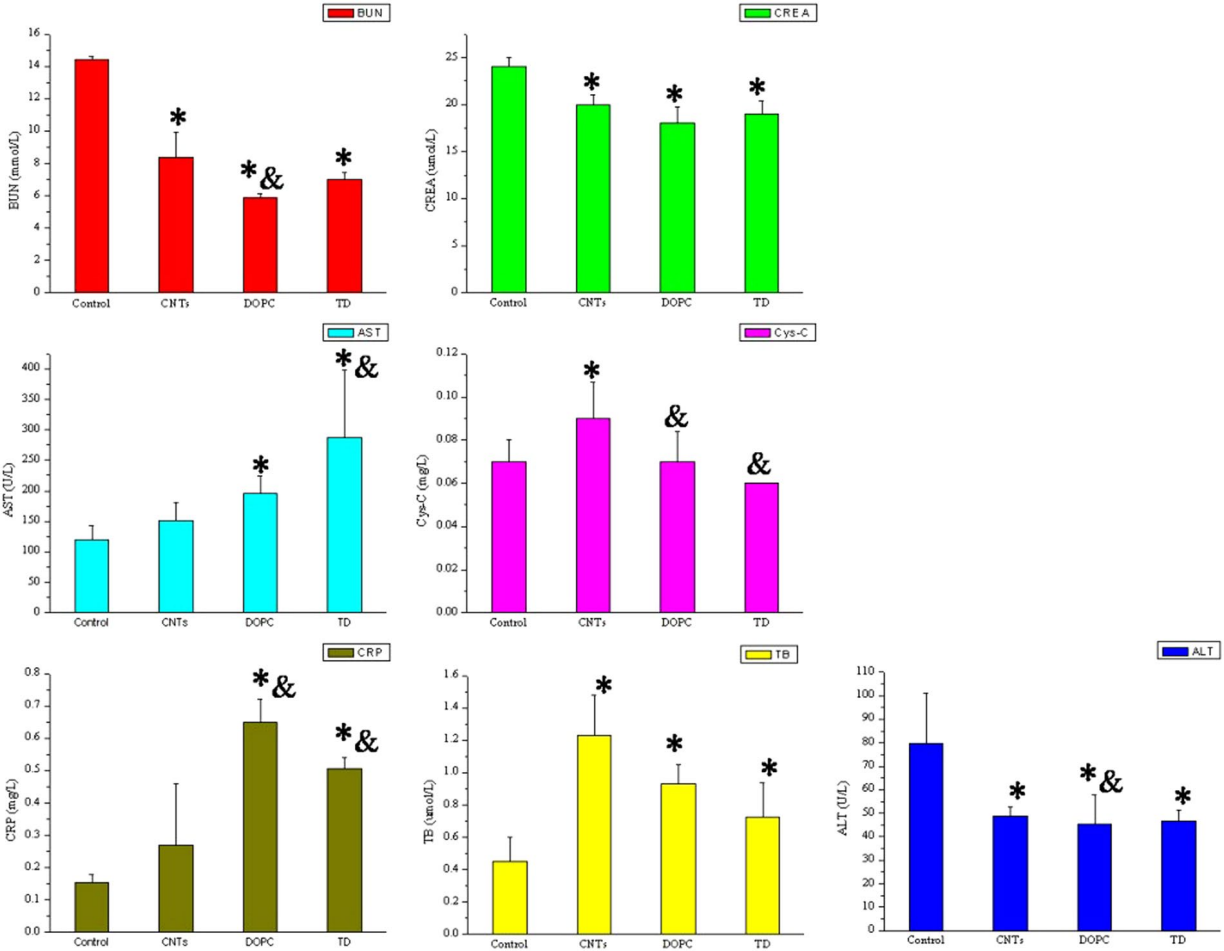
The biochemical indices level of BUN (blood urea nitrogen), CREA (creatinine), AST (aspartate aminotransferase), Cys-C (cystatin C), ALT (alanine aminotransferase), CRP (C-reaction protein) and TB (total bilirubin) change in plasma after exposure oMWCNTs to DOPC/or TD –model mice (*p < 0.05 for groups *vs*. control group; ^*&*^ *p* < 0.05 for groups vs. single oMWCNTs group; n = 5, +sem).

Compared with those in control group mice, the BUN, CREA, ALT, Cys-C and CRP levels in the plasma differed among the exposed groups of oMWCNTs, DOPC + oMWCNTs and TD + oMWCNTs (Fig. 6, *p < 0.05). However, the biochemical index contents, except for ALT and AST content, did not change in the exposed groups of DOPC + oMWCNTs and TD + oMWCNTs (Fig. 6). Compared with the pathological section of the control group mice (Fig. 5 and Figure S8), the lung in the exposed group of DOPC + oMWCNTs showed large amounts of red blood cells and severe haemorrhage. Early inflammatory response was observed, and the structure of hepatic tissues was damaged. The results also showed normal spleen and kidney, almost normal myocardial cells, and disorder cardiac muscle fibres. The exposed group of TD + oMWCNTs showed severely ruptured pulmonary alveoli and haemorrhage, and the hepatic cells were ruptured with a disordered structure. The morphology of the kidney tubules was ruptured with a slight haemorrhage, whereas the spleen and heart showed no obvious damages. At the same time, the browning of the nanoparticles was observed in all the tissue sections of the exposed groups. Overall, when mice were simultaneously exposed to DOPC/or TD with oMWCNTs, the pathological changes in the mice are consistent with those of the group exposed to oMWCNTs alone. These findings indicate that DOPC/or TD did not change the damaging effect of oMWCNTs *in vivo* after simultaneous co-exposure mice to DOPC/or TD and oMWCNTs.

**Figure 7:**
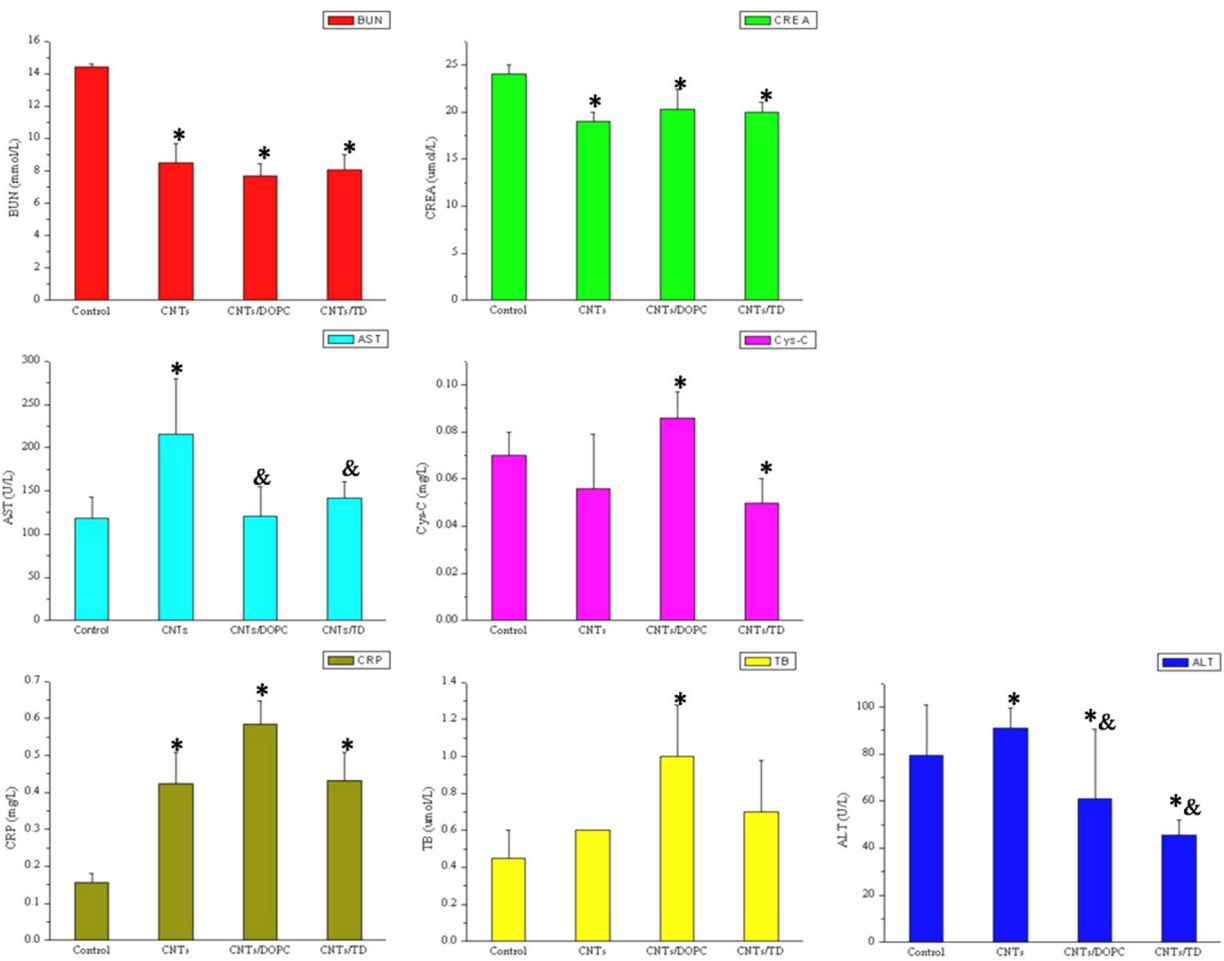
The biochemical indices level of BUN (blood urea nitrogen), CREA (creatinine), AST (aspartate aminotransferase), Cys-C (cystatin C), ALT (alanine aminotransferase), CRP (C-reaction protein) and TB (total bilirubin) change in plasma after exposure oMWCNTs and DOPC/or TD to mice simultaneously (*p < 0.05 for groups *vs*. control group; ^*&*^ *p* < 0.05 for groups vs. single oMWCNTs group; n = 5, +sem).

Mouse models of DOPC/or TD treatment were created to determine the preventive effect of these drugs on the damages of oMWCNTs. Figure 7 shows that the biochemical indices in the plasma of single-exposure oMWCNTs group or the DOPC/or TD-model mice exposed to oMWCNTs showed substantial difference compared with the control group of mice (Fig. 7, *p < 0.05). However, only a part of the biochemical indices of the DOPC/or TD-model mice exposed to oMWCNTs differed from those of mice exposed to oMWCNTs alone. Therefore, analysis of the biochemical indices indicated that the lack of prevention effect of DOPC/or TD on damages caused by oMWCNTs in mice. After exposing the DOPC-model mice to oMWCNTs, the pathological section showed that ruptured and bleeding pulmonary alveoli and obvious browning of the nanoparticles. The liver and kidney were nearly normal, the spleen nearly underwent fibrosis, and the cardiac muscle fibres became thin because of haemorrhage. The TD-model mice exposed to oMWCNTs possessed severely ruptured and bleeding of the pulmonary alveoli, destroyed central vein of liver and no obvious changes in the spleen. However, the kidney showed a slight haemorrhage and the gap of the cardiac muscle fibres increased without lesions. The pathological results further confirmed that the DOPC/or TD showed no preventive effects on the damages of oMWCNTs in mice (Fig. 7).

### Effect of TD dosage on treatment

**Figure 8:**
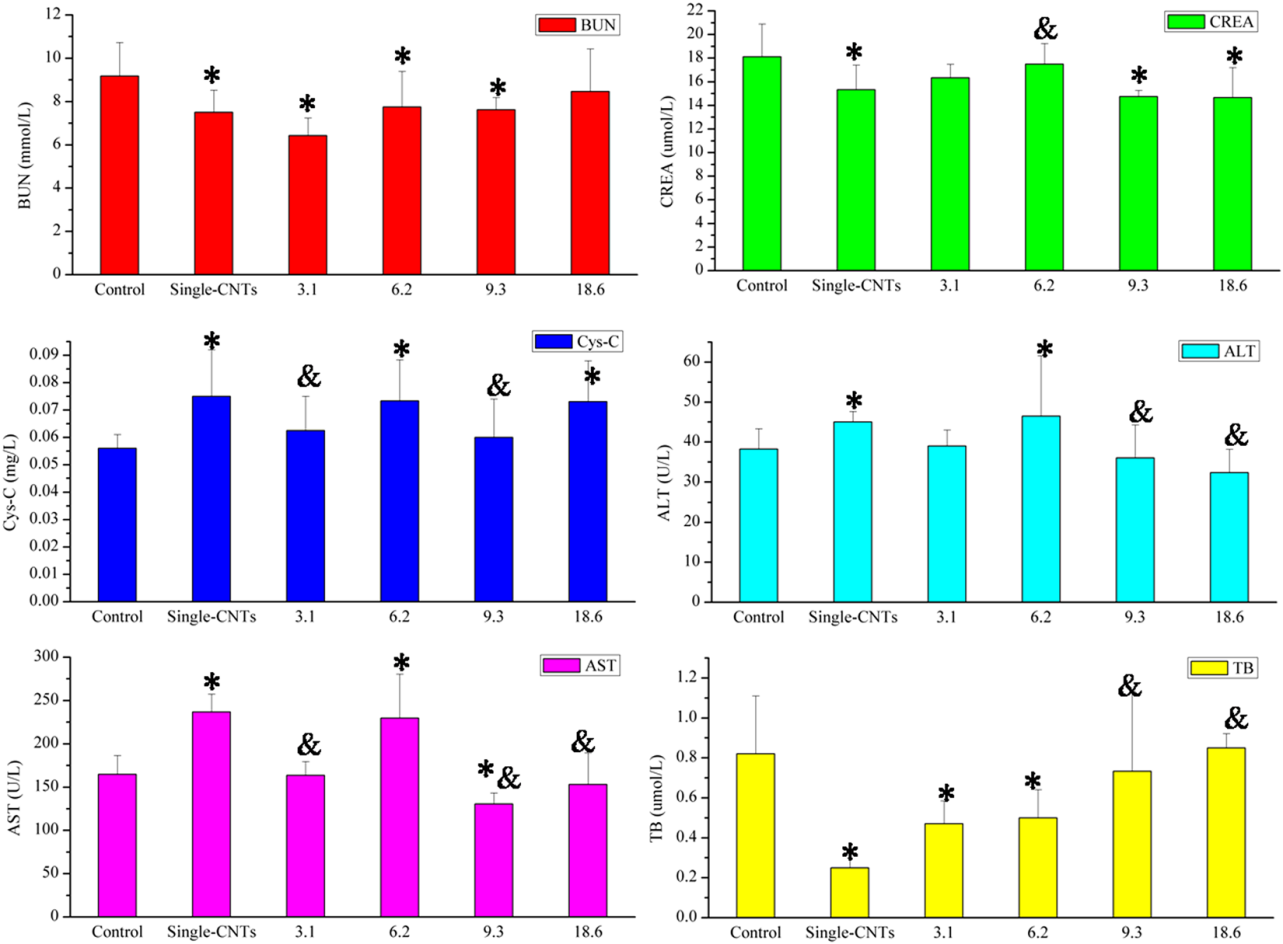
The biochemical indices level of BUN (blood urea nitrogen), CREA (creatinine), AST (aspartate aminotransferase), Cys-C (cystatin C), ALT (alanine aminotransferase), CRP (C-reaction protein) and TB (total bilirubin) change in plasma after exposure 3.1, 6.2, 9.3 and 18.6 mg/kg.bw TD to oMWCNTs-model mice (*p < 0.05 for groups *vs*. control group; ^*&*^ *p* < 0.05 for groups vs.single oMWCNTs group; n = 5, +sem).

The results showed that TD exhibited evident treatment effects on the damages caused by oMWCNTs on mice. Therefore, studying the effect of TD dosages on treatment is necessary. After exposing the oMWCNTs-model mice to about normal saline, 3.1, 6.2, 9.3 and 18.6 mg/kg.bw TD solutions, all mice were sacrificed to obtain the plasma. The biochemical indexes of the plasma such as BUN, CREA, Cys-C, ALT, AST and TB were measured by ELISA Kit. Compared with the results of the control group, various degrees of damages were observed after exposing mice to oMWCNTs; this confirms that the oMWCNTs-model mice were created successfully (Fig. 8, *p < 0.05). The contents of Cys-C, ALT, AST and TB in the plasma gradually increased to the normal levels as the oMWCNTs-model mice were exposed to TD (Fig. 8). Moreover, the inflammation related serum cytokine level before and after TD treatment in oMWCNTs-model mice were also detected to further prove the treatment effect of TD on the toxicity or damages of oMWCNTs in mice. As results showed, compared with control and single oMWCNTs group, the IL-10 and TNF-αlevels in serum recovered very well for TD exposure group (Figure S7).

### Effect of oMWCNTs and DOPC/or TD co-exposure on erythrocyte

**Figure 9:**
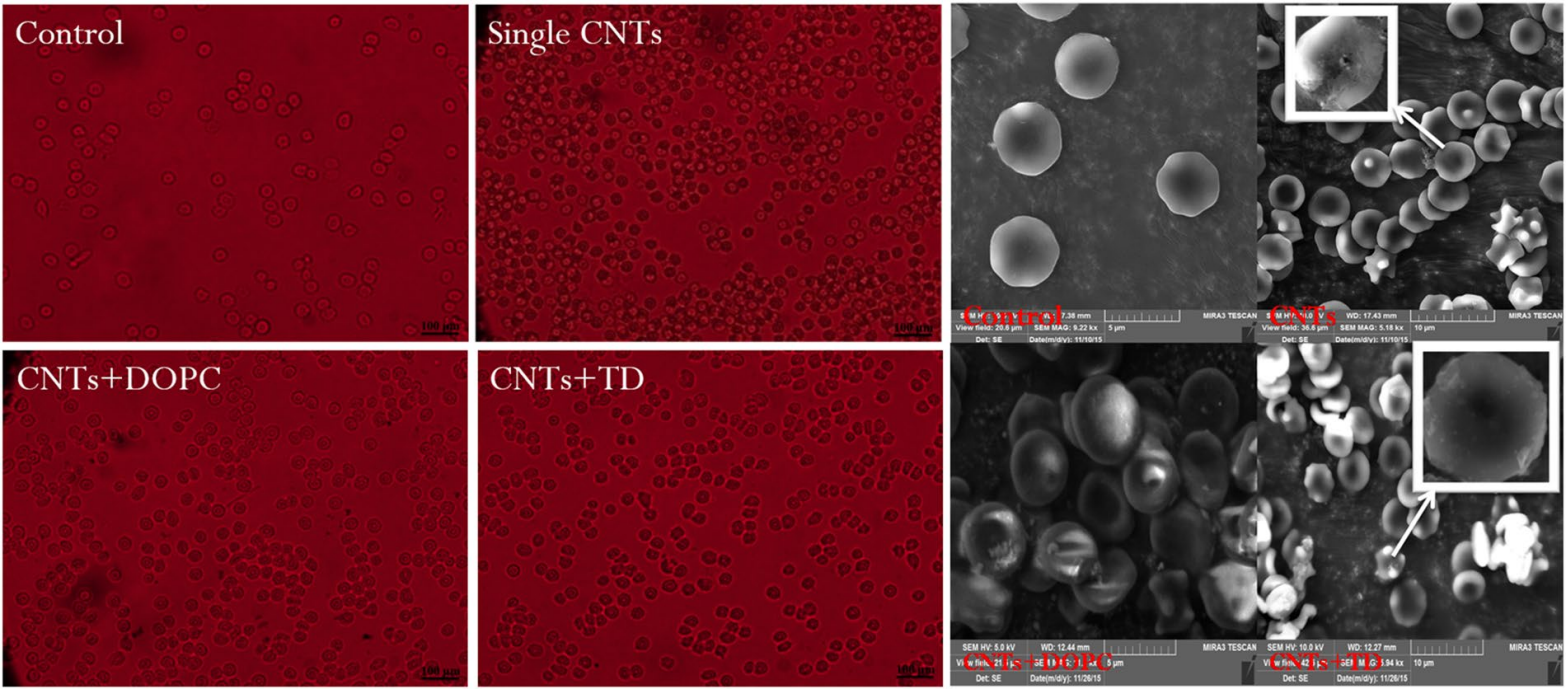
The cell morphology of erythrocytes under optical microscope (100×, scale bar is100 μm) and SEM (5–10 μm) after exposure saline solution, oMWCNTs, oMWCNTs + DOPC and oMWCNTs + TD to erythrocytes, respectively.

The results showed that altered and ruptured morphology of erythrocytes after incubation in oMWCNTs solution for about 2 h. The morphology of erythrocytes exhibited considerably changed after exposure to oMWCNTs with DOPC/or TD simultaneously (Fig. 9). Compared with control groups and single oMWCNTs groups (Figure S6), the viability of cells incubated in oMWCNTs and DOPC/or TD solution approximated the morphology of control groups. Figure 9 shows also the SEM images of damaged erythrocyte cell membranes after incubation in oMWCNTs solution compared with the control groups. The cell morphology was almost intact when the cells were exposed to oMWCNTs and DOPC/or TD simultaneously (Fig. 9). In addition, compared with the control groups, the blood routine results also showed that the model mice of oMWCNTs was created successfully, and the TD has a significant treatment effect on the damage of oMWCNTs in mice (Figures S9 and S10).

## Discussion

The effects of DOPC and/or TD on the biodistribution of oMWCNTs in mice were studied through ^131^I isotope tracer technique (Fig. 1). The labelling yields characterization of ^131^I-compounds were measured through paper chromatogram (Figure S4). According to the solubility property of Na ^131^I, DOPC/or TD and oMWCNTs in normal saline and acetone, the labelling yields of ^131^I-oMWCNTs, ^131^I-DOPC and ^131^I-TD measured through normal saline were 82.82%, 70.26% and 72.21%, respectively; and the labelling yields of ^131^I-DOPC and ^131^I-TD measured through acetone were 74.28% and 78.57%, respectively, which were all over 60%, and so the radio-tracing technique used in this paper could reflect the biodistribution and behaviour of ^131^I-compounds effectively *in vivo* ^38^. Briefly, after the intravenous of ^131^I-oMWCNTs, they loaded the venous blood from the right atrium into the left ventricle, the middle part of which included the pulmonary capillary bed. Most of ^131^I-oMWCNTs were captured by the pulmonary capillary bed and highly accumulated in the lungs. Some ^131^I-oMWCNTs in the lungs were eliminated and entered the circulatory system with time, whereas some were absorbed by macrophage organs (i.e., spleen, liver). As a result, high accumulation of ^131^I-oMWCNTs was observed in the spleen and liver (Fig. 1). At the same time, the accumulated of ^131^I-oMWCNTswould decrease with time going in tissues. These results are consistent with several previous reports^22, 37^. It could also be seen from the results that the presence of DOPC did not affect the distribution of ^131^I-oMWCNTs in mice. However, DOPC showed high biocompatibility, could be easily distributed evenly and excreted quickly upon entry into body without accumulating in the tissues^28^. Thus, oMWCNTs positively affected the distribution of ^131^I-DOPC in mice. In addition, when exposed oMWCNTs and TD to mice simultaneously, ^131^I-TD was mainly absorbed by the lung, liver and spleen of mice, which is significantly different from the previous reports about the TD distribution *in vivo* ^32–35^, but the distribution states were in accordance with that of oMWCNTs. These results showed that the presence of oMWCNTs considerably affected the distribution of TD in mice, but TD did not affect that of oMWCNTs.

What’ more, through synthesising the Ag/oMWCNTs nanocompounds, the influences of DOPC/or TD on biodistribution of oMWCNTs were further studied *in vivo* by improve the CT imaging. The experimental results confirmed the previous results. The dosage of TD did not cause obvious effects on the distribution of oMWCNTs *in vivo*. On the contrary, different dosages of DOPC greatly affected the biodistribution of Ag/oMWCNTs in mice, as seen in the CT imaging results (Fig. 2). As well known, the main component of biological membranes is DOPC, which has substantial compatibility with biological membranes. Therefore, when the mice were exposed to the mixture of DOPC and Ag/oMWCNTs, DOPC improved the compatibility of Ag/oMWCNTs in the body. Part of Ag/oMWCNTs could be aggregated in tissues with abound membranes coated with DOPC, such as the intestinal tract. Therefore, the lung CT imaging of Ag/oMWCNTs decreased as the mixture dosage of DOPC increased. However, in the region with DOPC/or TD used in this experiment, the biodistribution of Ag/oMWCNTs was not evidently affected by co-exposure to DOPC/or TD. Moreover, the improved CT imaging technique allows the exploration of the behaviour of nanoparticles *in vivo*.

Clinically, statins were mainly used to cure cardiovascular and cerebrovascular diseases for their efficacy of regulating blood lipids, anti-thrombosis, anti-inflammatory and others^29–31^ and might improve the biocompatibility of nanoparticles to promote their excretion from the body^39–41^. As a fat-soluble organic matter and the basic component of the cell membrane, DOPC is generally found in the tissues of animals and plants. DOPC could combine with carbon nanomaterials *in vitro* and form a layer of coating on the surface of carbon nanomaterials^28^. Therefore, the effect of DOPC/or TD on the toxicity of oMWCNTs to cells *in vitro* should be investigated firstly. Herein, the biocompatibility of oMWCNTs to erythrocytes affected by DOPC/or TD were evaluated by optical microscopy. The finding implies that the DOPC/or TD could improve the biocompatibility of oMWCNTs, but the DOPC exhibited better ability than that of TD. Furthermore, the influence of oMWCNTs on erythrocytes after simultaneous exposure to DOPC/or TD showed that the cytotoxicity of oMWCNTs was reduced in the presence of DOPC/or TD (Fig. 9). As our thought that the DOPC/or TD could be adsorbed onto oMWCNTs with a saturated adsorption (Figure S5). In addition, the DOPC/or TD are fat-soluble organic matter. The significantly different cell responses after exposure to oMWCNTs and DOPC/or TD might be ascribed to the different solubility of DOPC/or TD, resulting different biocompatibility of oMWCNTs on erythrocytes *in vitro*. However, the detailed mechanism for this phenomenon should be further studied in the future.

The effect of DOPC/or TD on the toxicity of oMWCNTs in mice showed that TD exhibited substantial therapeutic effect on the damages of oMWCNTs in mice, which is supported by previous investigations (Figs 4, 6, 7 and Figure S7). DOPC could coat the oMWCNTs released in the blood to improve their biocompatibility and decrease their toxicity when the oMWCNTs-model mice are exposed to DOPC/or TD, those conclusions were confirmed by the experiment of effect of oMWCNTs and DOPC/or TD co-exposure on erythrocyte (Fig. 9 and Figure S5); however, the damages and inflammation caused by oMWCNTs cannot be alleviated nor eliminated. By increasing the tissue gap (Fig. 5), TD could promote the excretion of nanoparticles from the body and shortened their accumulation time, so as to decrease the damages of oMWCNTs in mice. As a result, the biochemical indexes in the plasma returned to normal and the lesions were reduced or eliminated in pathological sections (Figs 4 and 7). Compared with the control group of mice, minimal pathological changes were observed in the spleen and kidney for the mice exposed to DOPC/or TD, but obvious pathological changes were observed in the heart. Upon simultaneous exposure to DOPC and oMWCNTs, DOPC improved the biocompatibility and even changed the biodistribution of oMWCNTs to reduce their damaging effects to the liver, spleen and kidney (Fig. 2). However, a portion of oMWCNTs could enter the heart and cause myocardial cell damage when biocompatibility is improved. The lung was still the main tissue where oMWCNTs accumulated, and substantial pathological changes were observed. TD exhibited a relatively poor effect on the damages of oMWCNTs in mice after co-exposure because of its small molecular structure. As shown in previous works, exposing DOPC/or TD-model mice to oMWCNTs resulted in no obvious differences in pathological lesions compared with those exposed to oMWCNTs alone. This finding illustrates that DOPC/or TD did not prevent the damages of oMWCNTs in mice. In addition, the increasing gap of cardiac muscle fibres with a normal cell structure after exposure to TD may have contributed to the drug effect of TD^31, 40^. Therefore, TD exhibited a considerable therapeutic effect on the damages caused by oMWCNTs on mice, and exposure to high dosages of TD resulted in more obvious therapeutic effects in the range of the experimental dosages. But the detailed therapeutic mechanism is not enough clear and should be further studied in the future.

## Conclusion

DOPC/or TD did not affect the biodistribution of oMWCNTs, which in turn, considerably influenced the distribution and metabolism of both drugs in mice. DOPC/or TD did not exhibit obvious preventive effects on damages caused by oMWCNTs, but TD significantly reduced the damages of oMWCNTs to the mice. Moreover, the therapeutic effect depended on the dosage of TD within the range of experimental dosages. Therefore, TD exhibited a good therapeutic effect on the injuries caused by oMWCNTs in mice. This work shows that the damages of nanoparticles *in vivo* could be inhibited effectively by seeking and exposing them to appropriate drugs. this study provides new insights and approaches for management of nanoparticles hazards.

## Methods

### Materials

MWCNTs were prepared through chemical vaporization deposition and were commercially obtained from Shenzhen Nanotech Port Co. Ltd., Guangdong, China. According to the product specification, the as-received MWCNTs were subjected to transmission electron microscopy (TEM) analyses. The particles had 10 μm length and 10 to 30 nm diameter, and purity of >96% (wt.%); the particles contained <3% amorphous carbon and <0.2% ash. The pristine MWCNTs (untreated MWCNTs) were added to 3 mol/L HNO_3_ solution to remove the hemispherical caps of the nanotubes^42^. The mixture containing 3 g of MWCNTs and 400 mL of 3 mol/L HNO_3_ was stirred ultrasonically for 24 h. The suspension was filtered and rinsed with de-ionised water until the pH of the suspension reached 6. The suspension was then dried at 80 °C. The treated MWCNTs (named oMWCNTs) were calcined at 450 °C for 24 h to remove the amorphous carbon. The purified oMWCNTs were characterised by TEM, FTIR spectroscopy and Raman spectroscopy analyses and were used to prepare 5 g/L suspension. 1,2-Dioleoyl-sn-glycero-3-phosphocholine (DOPC, purity >99%) was bought from Xi’an Ruixi Biological Technology Co., Ltd, and TD (purity over 99%) was purchased from Nanjing Ailikaide Chemistry Co., Ltd. All other reagents were analytical pure.

### ^131^I labelling method and yield measurement

Na ^131^I (about 1 mCi) was provided by the People’s Hospital of Gansu Province. According to the literature^38^, chloramine-T method was used to label oMWCNTs, DOPC and TD with Na ^131^I. The labelled mixtures were centrifuged at 12000 rpm for 10 min to purify ^131^I-oMWCNTs, and the supernatant was discarded to remove free Na ^131^I. The labelled mixtures were placed in a separation column to remove free Na ^131^Ito purify ^131^I-DOPC and ^131^I-TD (Sephadex G-25, the eluent was normal saline and speed was 0.5 mL/min with 1 mL as a tube, and counted the radioactive count of the tubes. And then the eluent with relative high radioactive count was collected to stand-by.) The yields of the labelled compounds were measured using a paper chromatogram with chromatographic solutions containing normal saline and acetone. If the labelling yield was over 60%, the labelled compounds could effectively reflect their distribution and metabolism *in vivo* (Figure S4 in Supplementary Information).

### Tissue distribution study

Kunming mice (female), with initial weights of 15–18 g, were provided by the Laboratory Centre for Medical Science, Lanzhou University, Gansu, China. All animals were housed in individual cages with controlled environment at 21 °C to 22 °C and lights on from 08:00 to 20:00 h. The mice were fed with food and tap water *ad libitum*. All animal protocols were in accordance with the European Communities Council Directive of November 24, 1986 (86/609/EEC) and approved by Institutional Animal Care and Use Committees of Gansu Province Medical Animal Center and Lanzhou University Animal Committees Guideline (China).The mice were grouped randomly (five mice/group) before the experiment. ^131^I-oMWCNTs + DOPC, oMWCNTs + ^131^I-DOPC, ^131^I-oMWCNTs + TD and oMWCNTs + ^131^I-TD (dose (1:1) 12.4 mg/kg.bw) solutions were injected intravenously into different groups of mice. The animals were sacrificed at 1, 6, 16 and 24 h post injection; heart, lung, liver, spleen and kidney tissues were immediately dissected. About 1–2 mL of blood was also collected. Each tissue was wrapped in foil, weighed and counted for ^131^I. The interactions between oMWCNTs and DOPC /or TD in mice were also investigated.

### Preparation of Ag/oMWCNT heterostructures

Ag/oMWCNTs heterostructures were prepared through the reduction of Ag ions in the oMWCNTs dispersion solution by using a previously reported method^36^. Briefly, AgNO_3_ solution (2 mL, 0.01 mol/L) was added to the oMWCNTs solution (20 mL, 0.156 g/L) under magnetic stirring for 30 min. An alkaline solution of sodium borohydride (pH = 9.5, 0.8 mg/L, 10 mL) was added to the oMWCNTs solution under vigorous stirring at room temperature for 2 h. A black dispersion of Ag/oMWCNTs was formed, and Ag/oMWCNTs heterostructures were injected into the mice for CT imaging (CT from Tongji Hospital, Discovery CT 750HD, GE healthcare. The GSI scan parameters were as follows: prep group for 30 s; scan mode was axial; gantry rotation speed was 0.5 s/circle; tube voltage was 140 and 80 kV; tube current was 630 mAs; detector coverage about 20 mm; noise index 15 combine with 30% ASIR; matrix size was 512 × 512; and slice thickness was 0.625 mm.). In this section, the mice were injected to Ag/oMWCNTs (about 12.4 mg/kg.bw) with different dosages of DOPC/or TD (6.2, 12.4 and 18.6 mg/kg.bw). Mice in the control group were exposed to normal saline. After 2 h, all mice were subjected to CT imaging. The effects of varying dosages of DOPC/or TD on the biodistribution of oMWCNTs in mice were further investigated through improved CT imaging.

### Effect of oMWCNTs dosages on mice

The mice were randomly divided into four groups (five mice/group). Three groups of mice were injected intravenously with 6.2, 18.6 and 31 mg/kg.bw of the oMWCNTs solution. The control group was treated with normal saline. Finally, the experimental mice were sacrificed at 24 h after exposure to oMWCNTs. Blood was collected, stored for 10 min at room temperature and was centrifuged at 4000 rpm to obtain the plasma. As well know, the liver is the main tissue for metabolism and detoxification of drugs in body, the induced hepatotoxicity is one of important and most common cases after exposure oMWCNTs to mice^43^. If there are damages or toxicity in tissues, the relative biochemical indices (such as C-reaction protein (CRP), alanine aminotransferase (ALT), aspartate aminotransferase (AST) and total bilirubin (TB)) will change in blood compared with normal group mice^44, 45^. The kidney plays an important role in drug excretion and detoxification. The most common used biochemical indices or proteins in blood are creatinine (CREA), blood urea nitrogen (BUN) or cystatin C (Cys-C), both assessing the functional status of the kidney and detecting specific damage^46^. And so, biochemical indices in the plasma were detected using ELISA Kit (Shanghai Elisa Biology Technology Co., Ltd.), reflecting the toxic effects of oMWCNTs on mice.

### Effects of DOPC and/or TD on the toxicity of oMWCNTs in mice

The following experimental processes were designed to study the prevention and treatment effects of DOPC and/or TD on the toxicity of oMWNCTs in mice:

1. Treatment effects: The oMWCNTs-model mice were obtained by intravenously injecting mice with 12.4 mg/kg.bw oMWNCTs on the first day. The oMWCNTs-model mice were randomly divided into three groups (five mice/group). The grouped mice were intravenously injected with 12.4 mg/kg.bw DOPC/or TD and with normal saline solution on the second and the third days, respectively. All mice were sacrificed on the fourth day, and blood was collected and stored for about 10 min at room temperature. The collected blood was centrifuged at 4000 rpm to obtain the plasma, which was stored in the refrigerator at about −20 °C.

2. Prevention effects: The normal mice were randomly categorised into three groups (about five mice/group). The mice were intravenously injected with 12.4 mg/kg.bw DOPC/or TD and normal saline on the first and second days, respectively. The mice were then exposed to 12.4 mg/kg.bw oMWCNTs on the third day. All mice were sacrificed on the fourth day. Blood was collected and stored for about 10 min at room temperature. The blood was then centrifuged at 4000 rpm to obtain the plasma, which was stored in the refrigerator at about −20 °C.

3. Synergistic effects: Normal mice were randomly categorised into three groups (five mice/group) and were intravenously injected with DOPC + oMWCNTs [(12.4 + 12.4) mg/kg.bw], TD + oMWCNTs [(12.4 + 12.4) mg/kg.bw] and normal saline. All mice were sacrificed at one day after the injection. Blood was collected and stored for about 10 min at room temperature. The blood was centrifuged at 4000 rpm to obtain the plasma, which was stored in a refrigerator at about −20 °C.

The concentrations of BUN, CREA, Cys-C, ALT, AST, TB and CRP in the plasma were obtained through the abovementioned processes and measured using an ELISA Kit. Mice from each group were dissected to collect heart, liver, spleen, lung and kidney tissues were collected. These tissues were fixed in 10% buffered formalin and processed for routine histology with haematoxylin and eosin staining by Shanghai Shunbai Biological Co., Ltd. The tissues were microscopically observed with an Olympus Microscope-CX41 (Olympus Corporation, Shinjuku, Tokyo, Japan) coupled with a digital camera. The extent of tissue injury was estimated semi-quantitatively and lesions scored as multi-focal fibrosis/or necrosis according to relative reports^47–49^, and the results were showed in Figure S8 (please see Supplementary Information).

### Dose effects of TD on treatment

On the first day, the mice were developed by intravenously injecting with 12.4 mg/kg.bw oMWCNTs as model. Then the mice were randomly categorised into five groups (five mice/group), and exposed intravenously to normal saline (as single oMWCNTs control group), 3.1, 6.2, 9.3 and 18.6 mg/kg.bw TD on the second and third days. Mice in the control group were treated with normal saline. All mice were sacrificed on the fourth day. Blood was collected, stored for about 10 min at room temperature and then centrifuged at 4000 rpm to obtain the plasma. The same biochemical indices of the plasma were determined using the above methods. In order to further prove the treatment effect of TD on the toxicity of oMWCNTs in mice, the serum cytokines levels of interleukin 6 (IL6), interleukin 10 (IL10) and tumor necrosis factor α (TNF-α) were carried out here. Briefly, two group mice (five to six mice/group) were exposed to 12.4 mg/kg.bw oMWCNTs solution intravenously at first day, then one group mice were exposed to 12.4 mg/kg.bw TD another one group mice were treated with normal saline at second and third days. In addition, the control group mice (five mice) were treated with normal saline at all the time. All mice were scarified on the fourth day. Blood was collected, stored for 24 h at 4 °C and then centrifuged at 4000 rpm to obtain serum. Then the cytokines of IL6, IL10 and TNF-α levels in serum were measured through ELISA Kit (by Noval-bio Co., Ltd.).

### Effect of co-exposure of oMWCNTs and DOPC/or TD on erythrocyte morphology

The effects of DOPC/or TD on the toxicity of oMWCNTs to erythrocyte suspension were also studied. Fresh blood was obtained from normal mice and anticoagulated with heparin. Anticoagulated blood was centrifuged at 4000 rpm for 5 min^50, 51^. The plasma and buffy coat were removed by aspiration. The separated erythrocytes were washed three times by centrifugation (4000 rpm, 5 min) in 10 volumes of saline solution. The supernatant and buffy coats of white cells were carefully removed with each wash. The washed erythrocytes were finally re-suspended to the desired hematocrit level using the same solution and stored at 4 °C. The effects of oMWCNTs, DOPC, TD, DOPC + oMWCNTs and TD + oMWCNTs (mass 1:1) on the morphology of erythrocytes were investigated by optical microscopy and scanning electron microscopy (SEM).

The erythrocyte suspension (10% hematocrit) was incubated with the solutions of oMWCNTs, DOPC, TD, DOPC + oMWCNTs and TD + oMWCNTs, respectively, and kept the content of exposure drugs about 0.5 g/L in the mixture solution. After 2 h, the reaction mixtures were dropped onto a glass slide and directly observed the morphology of erythrocytes affected under the presence of oMWCNTs, DOPC, TD, DOPC + oMWCNTs and TD + oMWCNTs through an optical microscopy. For SEM observations, the treated erythrocyte suspension was fixed with a glutaraldehyde solution (3%), centrifuged and washed with gradient ethanol. Finally, the samples were vacuum dried for use.

In order to study further the effect of DOPC/or TD on the damage of oMWCNTs in mice, the four groups mice were exposed to 12.4 mg/kg.bw oMWCNTs with different dosages of 0, 6.2, 12.4 and 18.6 mg/kg.bw DOPC/or TD intravenously as experimental groups, and the control groups mice were injected same volume saline. All mice were sacrificed at 24 h after various treatments, and blood was collected to measure the levels of white blood cell (WBC), red blood cell (RBC), hemoglobin (HGB), red blood cell specific volume (HCT), blood platelet (PLT) and plateletcrit (PCT) through blood routine examination.

### Statistical analysis

Statistical significance in the difference was carried out by analysis of variance (ANOVA) or Kruskal-Wails-test method after One-sample Kplmogorov-Smirnov Npar-test and Homogeneity of Variances test, and all statistical calculations were evaluated by SPSS software, and data were showed as the mean values + SD of multiple determinations.

## Electronic supplementary material

Supplementary Information
