# Supplementary material for: Curing the Toxicity of Multi-Walled Carbon Nanotubes through Native Small-molecule Drugs
Source: Sci Rep. 2017 Jun 6;7:2815. doi: 10.1038/s41598-017-02770-5 (PMC5460272; doi:10.1038/s41598-017-02770-5)
Supplement: Supplementary file 1 — Supplementary Information [file 41598_2017_2770_MOESM1_ESM.pdf]

Supplementary Information

Curing the Toxicity of Multi-Walled Carbon Nanotubes through Native Small-molecule Drugs

Wei Qi[1*$], Longlong Tian[2$], Wenzhen An[2$], Qiang Wu[2], Jianli Liu[3], Can Jiang[5], Jun Yang[6], Bing Tang[6], Yafeng Zhang[6], Kangjun Xie[1], Xinling Wang[7], Zhan Li[4*], Wangsuo Wu[2*]

[1]School of Chemistry and Chemical Engineering, Huazhong University of Science and Technology, Wuhan City, 430074, Hubei Province, P.R. China

[2]Lanzhou University, Lanzhou City, 730000, Gansu Province, P.R. China

[3]Lanzhou University Second Hospital, Lanzhou City, 730000, Gansu Province, P.R. China

[4]Lanzhou Institute of Chemical Physics, Chinese Academy of Sciences, Lanzhou, 730000, Gansu Province, P.R. China

[5]Non-power Nuclear Technology Research & Development Center, Hubei University of Science and Technology, Xianning City, 437000, Hubei Province, P.R. China

[6]Institue of Applied and Electromagnetic Engineering, Huazhong University of Science and Technology, Wuhan City, 430074, Hubei Province, P.R. China

[7]College of Pharmacy, Xinjiang Medical University, Urumqi, 830011, Xinjiang Province, P.R. China

[$]These authors contributed equally to this work.

[*]Correspondence and requests for materials should be addressed to ZL (lizhaner@126.com), QW (qiwei@hust.edu.cn) and WW(wuws@lzu.edu.cn).

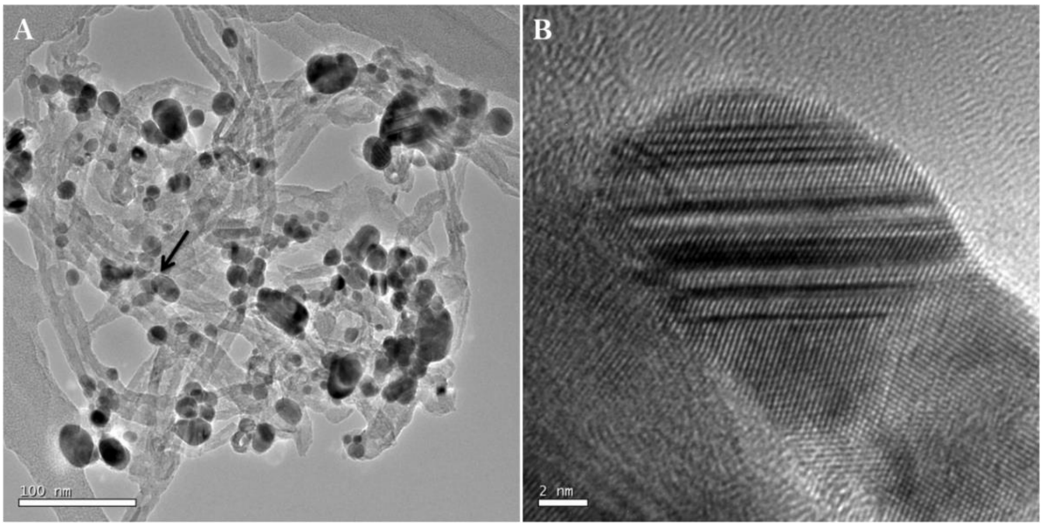

Figure S1 The TEM characterization of Ag/oMWCNTs.

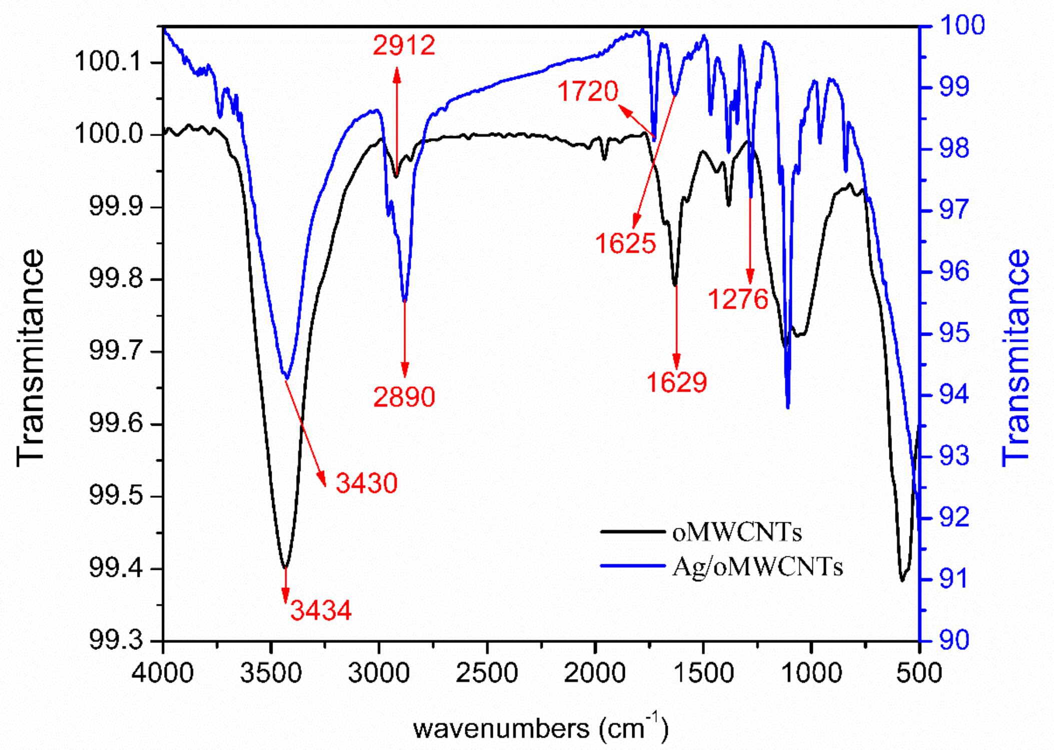

Figure S2 The FT-IR characterization of Ag/oMWCNTs and oMWCNTs.

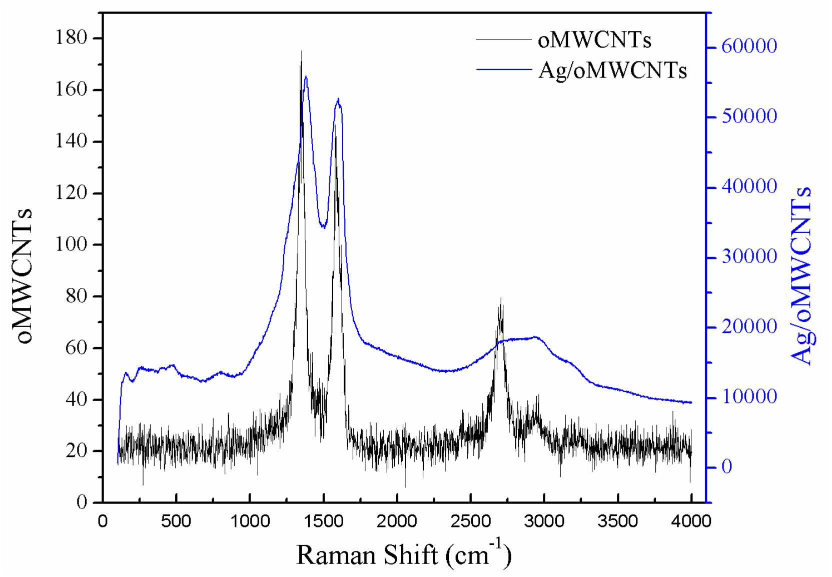

Figure S3 The Raman spectrum of Ag/oMWCNTs and oMWCNTs.

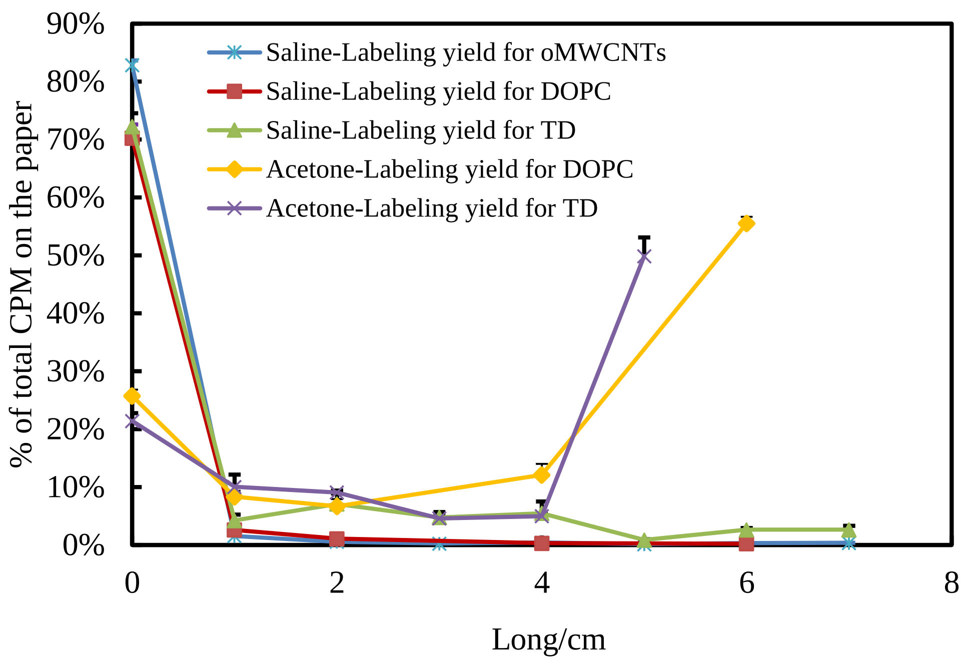

Figure S4 The labeling yields characterization of ^{131}I-compounds through paper chromatogram, the chromatographic solutions were normal saline and acetone. Note: In this section, A sample of the test solution (10 μL) was applied at 1 cm from the lower end of the strips (Whatman paper, Maidstone, Kent, UK). The strips were developed in normal saline and acetone until the solvent reached the top portion. The strips were dried and cut into 1-cm segments, and the distribution of radioactivity on the strip was determined using a gamma-ray counter. According to the solubility property of Na^{131}I, DOPC/or TD and oMWCNTs in normal saline and acetone, the labeling yields of ^{131}I-oMWCNTs, ^{131}I-DOPC and ^{131}I-TD measured through normal saline were 82.82%, 70.26% and 72.21%, respectively; and the labeling yields of ^{131}I-DOPC and ^{131}I-TD measured through acetone were 74.28% and 78.57%, respectively, which were all over 60%, and so the radiotracing technique used in this paper could reflect the biodistribution and behavior of ^{131}I-compounds effectively *in vivo*.

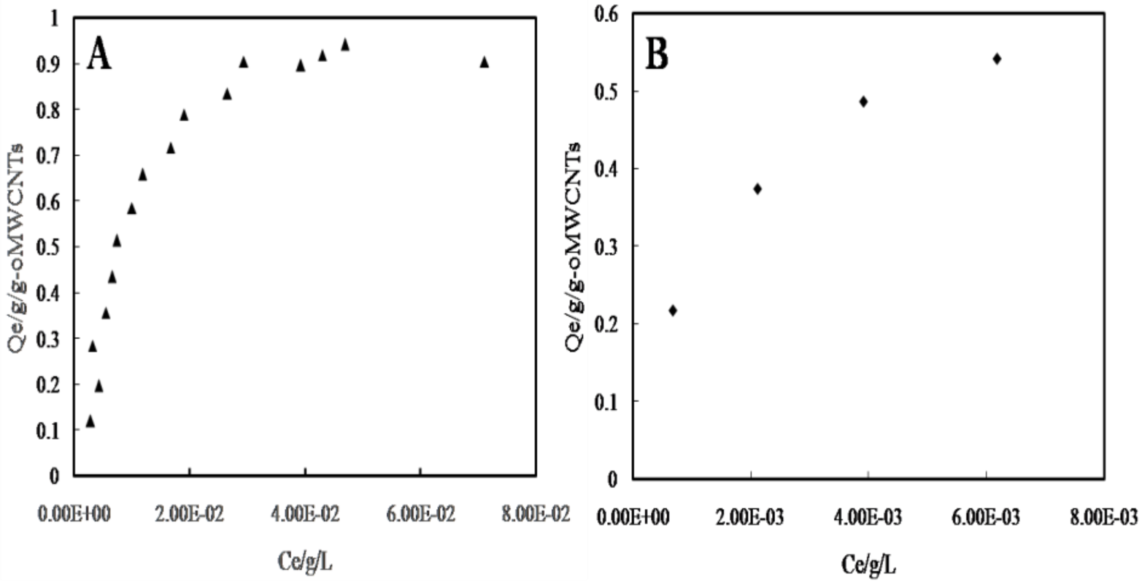

Figure S5 The adsorption isotherm of DOPC(A)/or TD (B)on the oMWCNTs (pH=7.3, t=37 ℃, I_{NaCl} =0.9%). Note: In this section, different concentrations of DOPC and TD were added into polythene centrifugal tubes with the 0.2 g/L oMWCNTs, and kept the final volume at 3mL. Then adjusting the pH by NaOH about 7.3 and ionic strength by NaCl about 0.9%, and shaking under 37 ℃ over 24 h until to reach equilibrium. Finally, the adsorption system was centrifuged at 12000 rpm about 20 min, and then collected the supernate. The content of DOPC and TD in precipitation and supernate was measured by Elemental Analyzer and Ultraviolet and visible spectrophotometer to investigate the adsorption capacity of DOPC/or TD onto oMWCNTs.

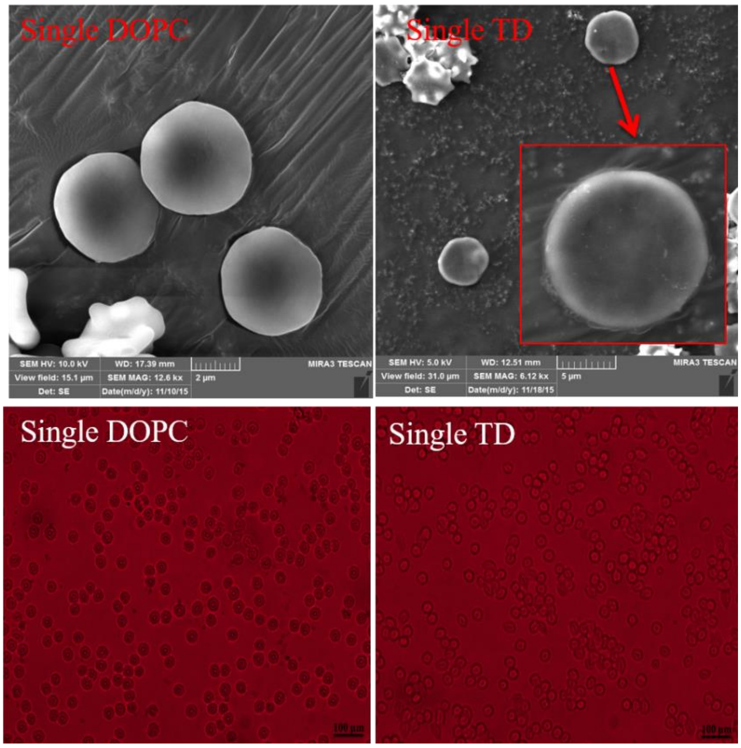

Figure S6 The cell morphology of erythrocytes under optical microscope (100×, scale bar is 100 μm) and SEM (5-10 μm) after exposure DOPC and TD, respectively.

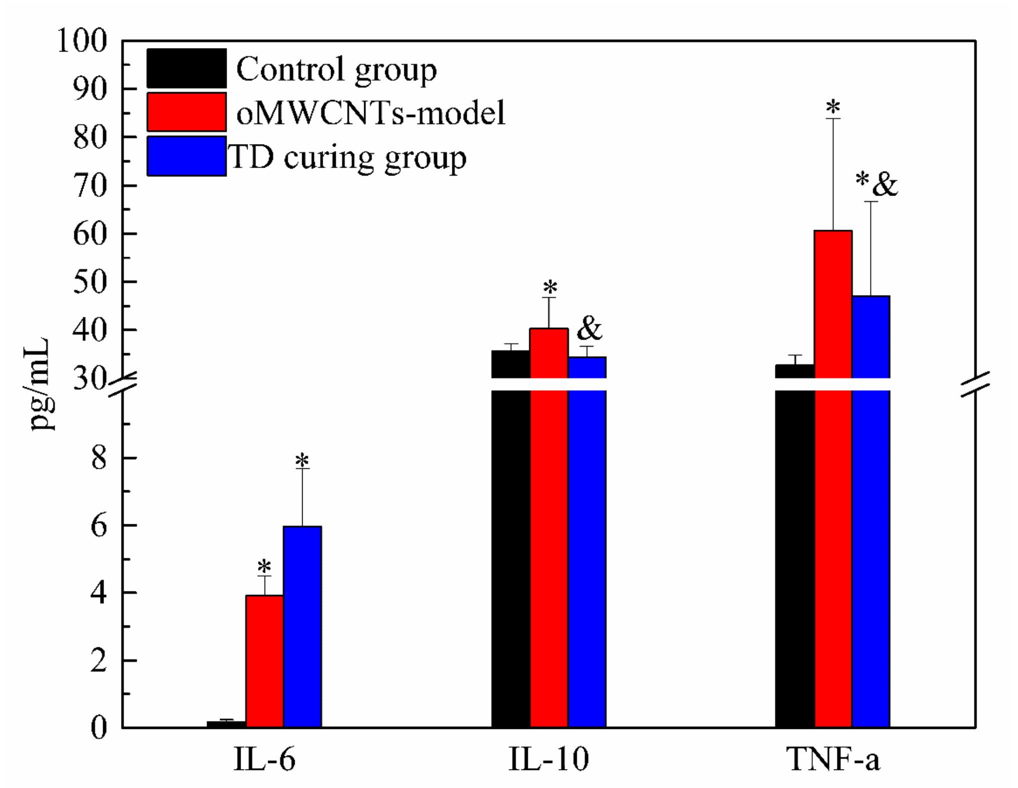

Figure S7 The cytokines of IL-6, IL-10 and TNF-α in serum before and after TD (12.4 mg/kg.bw) treatment in oMWCNTs-model mice ($^{*}p$<0.05 for groups *vs.* control group; $^{\&}p$<0.05 for groups vs. single CNTs group; n=5-6, +sem).

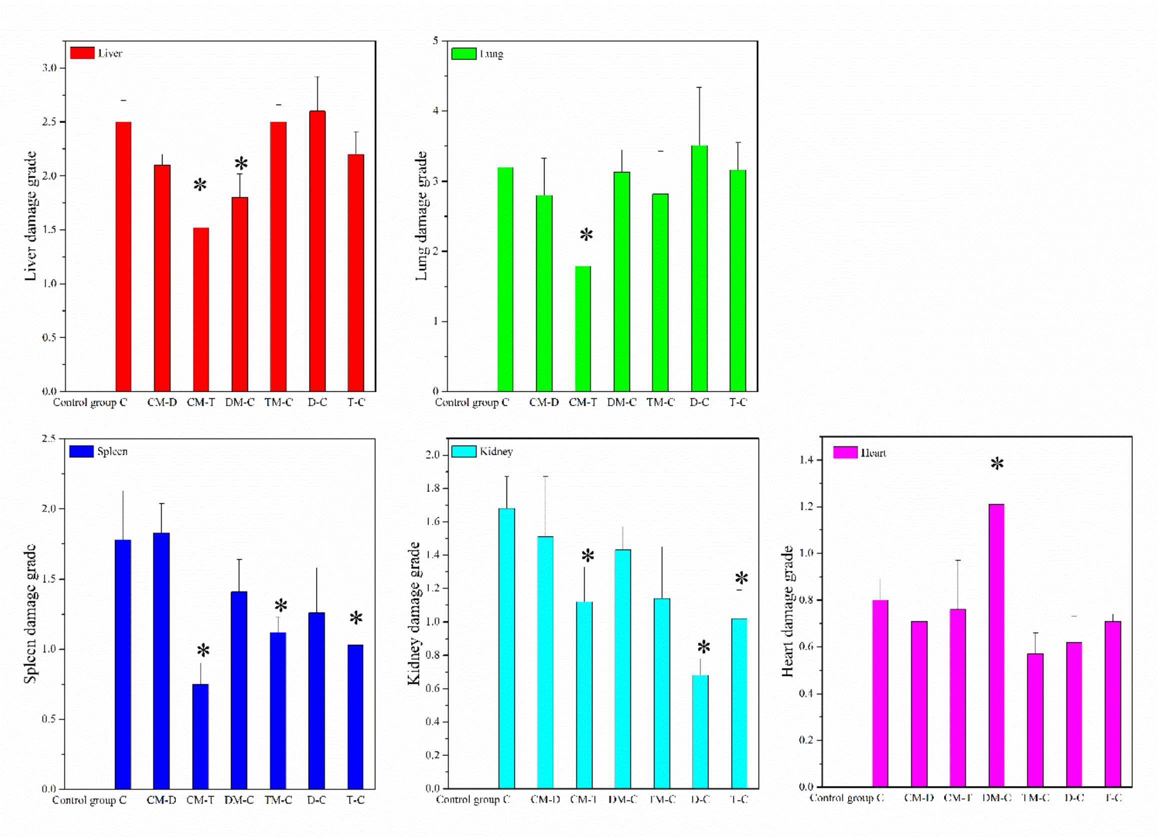

Figure S8 The morphological alterations in liver, lung, spleen, kidney and heart tissues after

exposure different drugs (C for the tissues of oMWCNTs-model group mice, CM-D for the tissues of the tissues of oMWCNTs-model +DOPC group mice, CM-T for the tissues of oMWCNTs-model +TD group mice, DM-C for the tissues of DOPC-model +oMWCNTs group mice, TM-C for the tissues of TD-model +oMWCNTs group mice, D-C for the tissues of DOPC+oMWCNTs group mice, T-C for the tissues of TD+oMWCNTs group mice, respectively. $^{*}p<0.05$ for groups *vs.* oMWCNTs-model group; n=5-6, +sem).

Note: In this experiment, the histology score of liver tissue was determined as Anita Patlolla *et al* [1] reported that 0= normal, 1 = mild cellular disruption in less than 25% of field area, 2 = moderate cellular disruption and hepato cellular vacuolation greater than 50% of field area, 3 = extensive cell disruption, hepato cellular vacuolation and condensed nuclei (pycknotic) of hepatocytes in greater than 50% of field area, 4 = extensive cell disruption, hepato cellular vacuolation, pycknotic and occasional central vein injury and 5= extensive cell disruption, multi central vein necrosis and degenerating of liver in more than 50% of field area. However, the assessment of histology scores of other tissues was determined as the following method:

Histology score	Present
0	Not present
1	This corresponds to a histologic change that may be barely noticeable to changes considered so minor, small, or infrequent as to warrant no more than the least assignable grade. For focal, multifocal or diffusely distributed lesions, this grade is used for processes where <10% of the tissue is involved. For hyperplastic/hypoplastic/atrophic lesions, this grade is used when the affected structure or tissue has undergone <10% increase or decrease in volume.
2	This corresponds to a histologic change that is a noticeable but not a prominent feature of the tissue. For focal, multifocal or diffusely distributed lesions, this grade is used for processes where between 10–39% of the tissue is involved. For hyperplastic/hypoplastic/atrophic lesions, this grade is used when the affected structure or tissue has undergone between an 10% and 39% increase or decrease in volume.
3	This corresponds to a histologic change that is a prominent feature of the tissue. For focal, multifocal or diffusely distributed lesions, this grade is used for processes where 40–79% of the tissue section is involved. For hyperplastic/hypoplastic/atrophic lesions, this grade is used when the affected structure or tissue has undergone between a 40% and 79% increase or decrease in volume.
4	This corresponds to a histologic change that is an overwhelming feature of the tissue. For focal, multifocal or diffusely distributed lesions, this grade is used for processes where 80–100% of the tissue section is involved. For hyperplastic/hypoplastic/atrophic lesions, this grade is used when the affected structure or tissue has undergone between a 80% and 100% increase or decrease in volume.

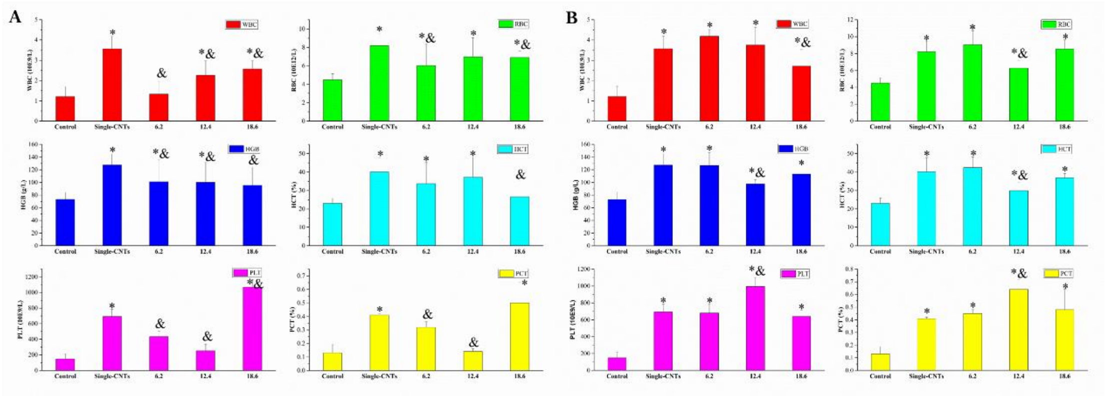

Figure S9 The level changes of white blood cell (WBC), red blood cell (RBC), hemoglobin (HGB), red blood cell specific volume (HCT), blood platelet (PLT) and plateletcrit (PCT) in blood after exposure 12.4 mg/kg.bw oMWCNTs with 6.2, 12.4, 18.6 mg/kg.bw TD (A) and DOPC (B) to mice (*p<0.05 for groups vs. control group mice; $^{\&}p$<0.05 for groups vs. oMWCNTs-model group; n=4, +sem).

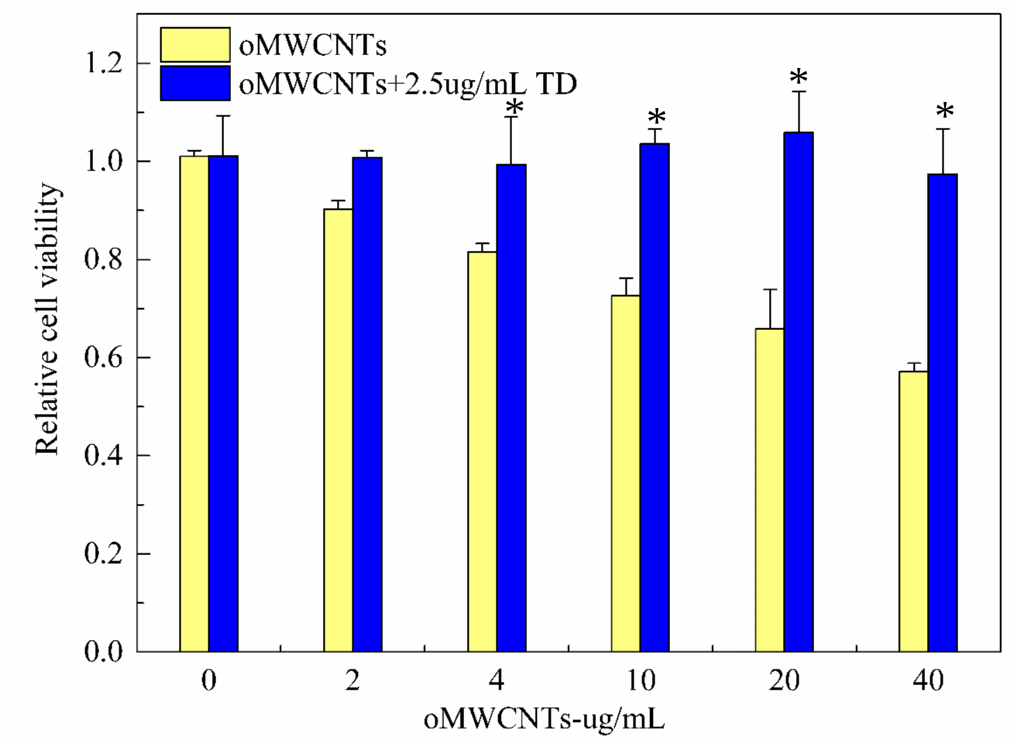

Figure S10 Relative viabilities of 4T1 cells at 24h after incubation with various dosages of oMWCNTs with 2.5 μg/mL TD (*p<0.05 for groups vs. single oMWCNTs group mice, n=10, +sem). (Note: In this experiment, 4T1 cells were incubated with 0, 2, 4, 10, 20 and 40 μg/mL single oMWCNTs or mixture with 2.5 μg/mL TD for 24h, then the relative cell viabilities were measured by the MTT assay, through which to further prove the anti-inflammatory effect of TD on the damage of oMWCNTs in mice.)

Reference:

1. Anita, P. et al. Biochemical and histopathological evaluation of functionalized single-walled carbon nanotube in Swiss-Webster mice. J Appl Toxicol 31, 75-83 (2011).
